# Artificial Intelligence in Healthcare and Public Health: Emerging Applications, Clinical Integration and Future Directions

**DOI:** 10.3390/bioengineering13050538

**Published:** 2026-05-06

**Authors:** Daniele Giansanti, Giovanni Costantini

**Affiliations:** 1 Centre IATIS, Istituto Superiore di Sanità, 000161 Rome, Italy; 2 Department of Electronic Engineering, University of Rome Tor Vergata, 00133 Rome, Italy; costantini@uniroma2.it

Artificial intelligence (AI) is increasingly reshaping the conceptual and operational foundations of public health [1]. Public health operates at the population level, requiring the integration of large-scale and heterogeneous data sources to support prevention, surveillance, and health system planning [1,2]. In this context, AI provides methodological tools capable of transforming complex data into actionable insights, with implications for both individual health interventions and population-level strategies [2].

Over recent years, the rapid expansion of digital health infrastructures—including electronic health records, mobile health applications, wearable devices, and connected monitoring systems—has further accelerated this transformation [2,3]. These technologies generate continuous streams of real-world data, creating new opportunities for modeling health dynamics but also introducing methodological and organizational challenges.

Within this evolving scenario, the present second edition of the Topic *“Artificial Intelligence in Public Health: Current Trends and Future Possibilities”* [4] builds upon the success of the first edition [5] by further consolidating the scientific discourse on AI applications in public health. The participation across multiple journals (*AI*, *Applied Sciences*, *Bioengineering*, *Clinics and Practice*, *Healthcare*, *International Journal of Environmental Research and Public Health*, *Journal of Clinical Medicine*, and *Journal of Imaging*) reflects the inherently interdisciplinary nature of this field and the need for contributions spanning engineering, clinical sciences, epidemiology, and health systems research.

Within this framework, the Topic is intended to provide a structured scientific space to support the advancement of knowledge on the role of artificial intelligence in public health [6,7,8,9,10]. In particular, it aims to facilitate a deeper understanding of how AI methodologies can be integrated within population health frameworks, digital health infrastructures, and healthcare systems to address emerging epidemiological, organizational, and technological challenges [7,9,10].

More broadly, this Topic reflects the growing recognition that the development of AI in public health requires continuous interdisciplinary exchange, combining methodological innovation with clinical insight, regulatory considerations, and public health expertise [6,7,10]. This perspective is essential to ensure that AI-driven approaches evolve in a way that is not only technically robust but also aligned with the principles of effectiveness, equity, and sustainability in population health systems [8,10].

This Topic includes 22 works (Contributions 1–22) together with this concluding editorial. To better frame the scope and implications of the contributions included in this Topic, (1) a cross-cutting synthesis is presented first, followed by (2) a detailed overview of each contribution and (3) a final synthesis highlighting the main implications of the Topic and outlining future directions for research and the responsible advancement of digital healthcare systems.

*Cross-cutting synthesis of the Topic*

Across the collected contributions (Contribution 1–22), a coherent and increasingly mature picture emerges of artificial intelligence (AI) as a transversal enabler of transformation across clinical practice, biomedical imaging, public health, and health system management. Rather than representing isolated applications, the studies collectively highlight a convergent evolution toward AI-augmented, data-driven, and patient-centered healthcare ecosystems.

A first cross-cutting domain concerns the integration of AI into diagnostic and clinical decision-support workflows, where multiple studies demonstrate a transition toward hybrid human–machine intelligence. Machine learning models for hospital-wide sepsis detection (Contribution 3), AI applications in electrocardiography for ischemic and structural heart disease (Contribution 5), AI-assisted left ventricular ejection fraction (LVEF) assessment using handheld ultrasound devices (Contribution 16), pediatric spine care applications (Contribution 9), and explainable AI approaches for ADHD prediction (Contribution 21) collectively highlight improvements in early detection, diagnostic precision, and interpretability.

A second transversal axis relates to the emergence of generative AI and natural language systems in patient education and clinical communication. Studies on AI chatbots in peritoneal dialysis education (Contribution 4), GPT-generated discharge instructions compared with clinician-written texts (Contribution 10), ChatGPT applications in chronic rhinosinusitis management (Contribution 17), and bibliometric analyses of generative AI in healthcare (Contribution 12) collectively show increasing integration of large language models into healthcare communication, with simultaneous opportunities for accessibility and concerns regarding reliability and governance.

A third cross-cutting dimension involves the expansion of AI-enabled digital health ecosystems and remote monitoring infrastructures, supporting a shift toward continuous and predictive healthcare models. AI-driven mobile health applications (Contribution 6), AI-enabled remote patient monitoring systems (Contribution 8), and machine learning approaches for predicting resilience among nurses (Contribution 18) illustrate this transition from episodic to longitudinal and preventive care.

A fourth thematic cluster concerns multimodal and next-generation biomedical data integration. Immersive and vision–language model tools in digital pathology (Contribution 2), the evolving role of radiation therapy technologists in head and neck cancer workflows (Contribution 1), and AI-based detection of autism traits using voice and behavioral data (Contribution 13) demonstrate the convergence of imaging, textual, and behavioral data streams into unified analytical frameworks.

A fifth dimension addresses system-level, ethical, and organizational implications of AI adoption in healthcare. Contributions on integrating AI into internal medicine and P6 medicine (Contribution 11), sustainability of AI-assisted mental health interventions (Contribution 14), risks and benefits of remote patient monitoring (Contribution 8), and AI in pediatric spine care ethics (Contribution 9) and AI clinical decision support systemacceptance and implementation factors (Contribution 22) collectively emphasize governance, safety, and long-term sustainability as central to implementation.

Finally, contributions on digital twins in oncology (Contribution 19) and population-level analyses such as COVID-19 impacts on life expectancy (Contribution 20) extend the scope of AI toward system modeling and macro-level health intelligence, highlighting its role in simulation, forecasting, and population health management.

*Detailed synthesis of the Topic contributions*

A coherent and increasingly mature picture emerges from the collected contributions (Contribution 1–Contribution 22), highlighting artificial intelligence (AI) as a transversal enabler of transformation across clinical practice, biomedical imaging, public health, education, and health systems. Rather than being limited to isolated technical demonstrations, the studies collectively reflect a progressive integration of AI into clinical workflows, decision-making processes, education, and health service organizations, with growing attention to real-world deployment, human factors, and ethical as well as organizational implications.

Within this landscape, Contribution 1 explores the evolving role of radiation therapy technologists in head and neck cancer care, showing a gradual shift from purely technical execution toward broader clinical involvement. The proposed operational framework positions technologists as active contributors to patient positioning, treatment delivery, monitoring, and supportive care, reflecting an increasingly interdisciplinary configuration of radiotherapy workflows.

Building on this clinical reconfiguration, Contribution 2 examines the concept of the “augmented cytopathologist,” integrating immersive technologies and vision–language models within digital pathology. It illustrates how VR/AR/XR environments and AI copilots may support diagnostic reasoning, spatial interpretation, and workflow efficiency, suggesting an emerging hybrid human–AI configuration in pathology practice.

Moving into acute care applications, Contribution 3 presents a hospital-wide machine learning model for sepsis detection, developed and validated on a prospectively expert-verified cohort. The study targets earlier identification of sepsis beyond conventional clinical scoring systems, with the aim of enabling more timely and accurate intervention in critical care settings.

In a complementary patient-facing perspective, Contribution 4 evaluates AI chatbots used in peritoneal dialysis education, comparing the quality, readability, and reliability of generated content. The findings indicate potential value in supporting patient education and self-management while also highlighting variability in output quality and the need for careful clinical validation of such tools. Contribution 5 then reviews AI applications in electrocardiography for ischemic and structural heart disease, synthesizing advances in machine learning-based ECG interpretation. It emphasizes improved diagnostic performance and reduced variability while also noting ongoing challenges related to generalizability and clinical translation.

Shifting toward digital health ecosystems, Contribution 6 analyzes recent advances in AI-driven mobile health (mHealth), underlining how smartphone-based and connected technologies, increasingly enhanced by AI, are reshaping healthcare delivery, continuous monitoring, and patient engagement in real-world contexts.

From a longitudinal and integrative perspective, Contribution 7 proposes an AI framework for sarcopenia prediction by linking mental health trajectories to physical risk. This introduces a dynamic view in which psychological patterns over time contribute meaningfully to predicting physical decline in aging populations.

In parallel, Contribution 8 examines AI-enabled remote patient monitoring systems, balancing opportunities and risks. While such systems can enhance accessibility and efficiency of care delivery, concerns remain regarding safety, equity, and data governance in continuous monitoring environments. Contribution 9 extends the discussion to pediatric populations, reviewing AI applications in pediatric spine care across clinical, research, and ethical dimensions. It highlights potential gains in diagnostic and treatment planning, alongside the need for cautious and responsible implementation in vulnerable groups.

Contribution 10 focuses on communication and patient perception, comparing GPT-generated and clinician-written discharge texts. It shows that while generative AI can support the production of patient-oriented communication, differences in empathy, wording, and style may significantly influence patient interpretation and trust. Contribution 11 situates AI within a broader conceptual shift in internal medicine through the “P6 medicine” framework. Here, AI is framed as an enabling layer for more personalized, predictive, preventive, participatory, and precision-oriented models of care. Contribution 12 provides a bibliometric mapping of generative AI in healthcare, illustrating the rapid expansion of the field and the emergence of structured research clusters. This reflects the consolidation of generative models as a central and fast-evolving research domain in medicine. Contribution 13 explores AI-based approaches for detecting autism traits using voice and behavioral data, focusing on computational phenotyping approaches that aim to support earlier and more objective identification of autism spectrum traits. Contribution 14 systematically reviews the sustainability of AI-assisted mental health interventions, assessing evidence on long-term usability, effectiveness, and scalability across mental health care systems. Contribution 15 examines the role of AI in enhancing healthcare for people with disabilities, highlighting improvements in accessibility, assistive technologies, and inclusive care pathways, while also acknowledging implementation and equity-related challenges. Contribution 16 evaluates AI-assisted left ventricular ejection fraction (LVEF) assessment using handheld ultrasound devices, showing promising alignment with reference standards and reinforcing the potential of point-of-care AI-supported cardiac evaluation. Contribution 17 investigates the use of ChatGPT in chronic rhinosinusitis with nasal polyps, critically assessing its potential as a clinical support tool while also underscoring limitations in reliability and domain-specific performance. Contribution 18 applies machine learning techniques to predict resilience among nurses, identifying key factors associated with psychological resilience and contributing to strategies aimed at workforce sustainability and mental health support. Contribution 19 reviews the emerging role of digital twins in cancer treatment, synthesizing how virtual patient models combined with AI can support simulation of disease progression and personalized treatment response in oncology. Contribution 20 analyzes the impact of the COVID-19 pandemic on life expectancy in South Korea, offering population-level insights into mortality changes and broader implications for public health systems. Contribution 21 focuses on explainable AI approaches for ADHD prediction, emphasizing interpretability as a critical requirement for clinical trust, transparency, and eventual adoption in mental health applications.

Finally, Contribution 22 investigates medical practitioners’ acceptance and use of AI-based clinical decision support systems in Western China, identifying key adoption barriers and facilitators through a mixed-methods approach and highlighting the central role of usability, awareness, trust, and organizational context in real-world implementation.

Taken together, these contributions outline a field that is steadily moving from isolated technological applications toward more integrated, context-sensitive, and human-centered AI systems in healthcare, in which innovation is increasingly shaped by clinical needs, organizational structures, and ethical considerations.

*Closing synthesis and lessons for the future*

Taken together, the contributions (Contribution 1–Contribution 22) outline a field that is moving beyond the exploratory phase of artificial intelligence (AI) in healthcare toward a more structured and practice-oriented stage of integration. Across very different clinical domains, a consistent direction emerges: AI is no longer positioned as a stand-alone technological add-on but increasingly as an embedded component of clinical reasoning, workflow organization, and health system design. This transition is visible both in high-acuity settings—such as sepsis detection, cardiology, and oncology—and in more distributed contexts, including patient education, remote monitoring, and mental health support.

At the same time, the collected evidence suggests that technological performance alone is no longer the central question. Instead, many studies converge on implementation as the key bottleneck. The work on clinical decision support adoption highlights how organizational readiness, awareness, and training can be more decisive than algorithmic capability itself. Similarly, studies on chatbots, generative AI, and remote monitoring systems underline that usability, trust, interpretability, and perceived reliability strongly shape real-world uptake. In this sense, the literature collectively shifts attention from “what AI can do” to “how, where, and under which conditions it is actually used.”

Another cross-cutting theme is the gradual expansion of AI from diagnostic support toward broader socio-technical functions. Several contributions illustrate this trajectory: from decision support in acute care and imaging to patient-facing communication tools to workforce resilience prediction and system-level modeling. This reflects an emerging view of AI as a transversal infrastructure for healthcare systems, rather than a set of isolated applications. In parallel, there is increasing attention to hybrid configurations in which human expertise and machine intelligence are explicitly co-designed, as seen in augmented pathology, explainable models, and AI-assisted imaging.

Importantly, ethical, interpretability, and human-factor considerations are no longer peripheral. They are increasingly central to how AI systems are evaluated. Explainable AI for mental health prediction, studies on patient preferences for AI-generated communication, and analyses of risks in remote monitoring all point toward a maturing awareness that clinical acceptability depends on transparency, accountability, and alignment with patient and professional values.

From a public health perspective, the contributions also indicate a widening scope: AI is being applied not only to individual diagnosis and treatment but also to population-level prediction, workforce sustainability, disability inclusion, and health system resilience. This broader framing suggests a gradual convergence between clinical AI and digital public health, where shared data infrastructures and predictive models support both individual and collective health outcomes.

Overall, a coherent set of lessons emerges. First, successful AI integration depends less on isolated model performance and more on ecosystem readiness, including governance, training, and workflow redesign. Second, human-centered design is becoming a prerequisite rather than an option, particularly in patient-facing and decision-support tools. Third, the field is moving toward multi-level integration, where AI simultaneously operates at the levels of data interpretation, clinical decision-making, and system organization. Finally, the need for rigorous real-world validation remains a defining challenge before scalable deployment can be fully achieved.

In conclusion, the collected contributions portray a discipline in transition: from innovation-driven experimentation to structured clinical and systemic integration. The trajectory is clear, but not linear, and its consolidation will depend on the ability to align technological advancement with human, organizational, and ethical dimensions of care.

## References

[1] Olawade D.B., Wada O.J., David-Olawade A.C., Kunonga E., Abaire O., Ling J. (2023). Using artificial intelligence to improve public health: A narrative review. Front. Public Health.

[2] Fernandes Prabhu D., Gurupur V., Stone A., Trader E. (2025). Integrating Artificial Intelligence, Electronic Health Records, and Wearables for Predictive, Patient-Centered Decision Support in Healthcare. Healthcare.

[3] de-la-Fuente-Robles Y.-M., Ricoy-Cano A.-J., Albín-Rodríguez A.-P., López-Ruiz J.L., Espinilla-Estévez M. (2022). Past, Present and Future of Research on Wearable Technologies for Healthcare: A Bibliometric Analysis Using Scopus. Sensors.

[4] https://www.mdpi.com/topics/7M3GWF5N7E.

[5] https://www.mdpi.com/topics/C52Z967WA3.

[6] Pashkov V.M., Harkusha A.O., Harkusha Y.O. (2020). Artificial Intelligence in Medical Practice: Regulative Issues and Perspectives. Wiad. Lek..

[7] Yousefi F., Dehnavieh R., Laberge M., Gagnon M.P., Ghaemi M.M., Nadali M., Azizi N. (2025). Opportunities, challenges, and requirements for Artificial Intelligence (AI) implementation in Primary Health Care (PHC): A systematic review. BMC Prim. Care.

[8] Dankwa-Mullan I. (2024). Health Equity and Ethical Considerations in Using Artificial Intelligence in Public Health and Medicine. Prev. Chronic Dis..

[9] Karami M., Madlool H. (2025). Artificial intelligence and digital health in the health systems of developing countries: The challenges and vision of integration in the primary health care setting. Front. Digit. Health.

[10] Gao Q., Chen L., Huang Z. (2026). Opportunities and challenges of artificial intelligence in public health: A systematic review on technological efficacy, ethical dilemmas, and governance pathways. Front. Public Health.

